# Upregulation of CCNB2 and Its Perspective Mechanisms in Cerebral Ischemic Stroke and All Subtypes of Lung Cancer: A Comprehensive Study

**DOI:** 10.3389/fnint.2022.854540

**Published:** 2022-07-19

**Authors:** Ming-Jie Li, Shi-Bai Yan, Gang Chen, Guo-Sheng Li, Yue Yang, Tao Wei, De-Shen He, Zhen Yang, Geng-Yu Cen, Jun Wang, Liu-Yu Liu, Zhi-Jian Liang, Li Chen, Bin-Tong Yin, Ruo-Xiang Xu, Zhi-Guang Huang

**Affiliations:** ^1^Department of Pathology/Forensic Medicine, The First Affiliated Hospital of Guangxi Medical University, Nanning, China; ^2^Department of Neurology, The First Affiliated Hospital of Guangxi Medical University, Nanning, China; ^3^Department of Neurology, Liuzhou People’s Hospital, Liuzhou, China; ^4^The Seventh Affiliated Hospital of Guangxi Medical University, Wuzhou Gongren Hospital, Wuzhou, China; ^5^Department of Gerontology, No. 923 Hospital of Chinese People’s Liberation Army, Nanning, China

**Keywords:** cyclin B2, cerebral ischemic stroke, lung cancer, single-cell RNA-seq, co-expression

## Abstract

Cyclin B2 (CCNB2) belongs to type B cell cycle family protein, which is located on chromosome 15q22, and it binds to cyclin-dependent kinases (CDKs) to regulate their activities. In this study, 103 high-throughput datasets related to all subtypes of lung cancer (LC) and cerebral ischemic stroke (CIS) with the data of CCNB2 expression were collected. The analysis of standard mean deviation (SMD) and summary receiver operating characteristic (SROC) reflecting expression status demonstrated significant up-regulation of CCNB2 in LC and CIS (Lung adenocarcinoma: SMD = 1.40, 95%CI [0.98–1.83], SROC = 0.92, 95%CI [0.89–0.94]. Lung squamous cell carcinoma: SMD = 2.56, 95%CI [1.64–3.48]. SROC = 0.97, 95%CI [0.95–0.98]. Lung small cell carcinoma: SMD = 3.01, 95%CI [2.01–4.01]. SROC = 0.98, 95%CI [0.97–0.99]. CIS: SMD = 0.29, 95%CI [0.05–0.53], SROC = 0.68, 95%CI [0.63–0.71]). Simultaneously, protein-protein interaction (PPI) analysis indicated that CCNB2 is the hub molecule of crossed high-expressed genes in CIS and LC. Through Multiscale embedded gene co-expression network analysis (MEGENA), a gene module of CIS including 76 genes was obtained and function enrichment analysis of the CCNB2 module genes implied that CCNB2 may participate in the processes in the formation of CIS and tissue damage caused by CIS, such as “cell cycle,” “protein kinase activity,” and “glycosphingolipid biosynthesis.” Afterward, *via* single-cell RNA-seq analysis, CCNB2 was found up-regulated on GABAergic neurons in brain organoids as well as T cells expressing proliferative molecules in LUAD. Concurrently, the expression of CCNB2 distributed similarly to TOP2A as a module marker of cell proliferation in cell cluster. These findings can help in the field of the pathogenesis of LC-related CIS and neuron repair after CIS damage.

## Introduction

The etiology of cerebral ischemic stroke (CIS) is complex and diverse. The traditional high-risk factors include hypertension, hyperlipidemia, and diabetes, among others (Du et al., 2021). Recently, there have been increasing reports of CIS caused by malignant tumors (Salazar-Camelo et al., 2021; Wu et al., 2021b). Tumors disrupt organ function and affect the stability of the body’s internal environment. Furthermore, the radiotherapy and chemotherapy drugs and surgery used in tumor treatment also significantly impact the body (Sooklal and Siddiki, 2021). Some retrospective studies have reported a risk of CIS in patients with malignant tumors, such as prostate cancer, head and neck tumors, colorectal cancer, and cervical cancer, and most of these patients have no traditional CIS risk factors (Aspberg et al., 2020; Ciolli et al., 2021), suggesting that the occurrence of CIS in some patients with malignant tumors is unrelated to the pathogenesis of traditional cerebrovascular diseases but is directly or indirectly related to tumors, which is called tumor-related CIS. Lung cancer (LC) is one of the tumors with the highest incidence worldwide, and its death rate is increasing. Even in some developing countries, LC is the malignant tumor most frequently occurring. Studies have confirmed the association between LC patients and increased risk of subsequent stroke (Chen et al., 2011; Anai et al., 2022). However, the detailed and incisive mechanism is still unclear. Therefore, clarifying the common pathogenesis of LC and CIS will be of great significance to the studies on the correlation between CIS and LC by providing them with a new theoretical perspective and actively promoting interdisciplinary research.

Recent studies have found that an uncontrolled cell cycle is a significant cause of unordered cell proliferation and carcinogenesis. Meanwhile, numerous experimental animal models confirmed the abnormal expression of cyclin in neurons after ischemia. Therefore, CCNB2 (CCNB2) was selected as our research focus. CCNB2 belongs to type B cell cycle family protein, which is located on chromosome 15q22, and it binds to cyclin-dependent kinases (CDKs) to regulate their activities (Wu et al., 2021a; Xia T. et al., 2021). Different cyclins have different spatiotemporal specificities at specific stages of the cell cycle (Gupta R. et al., 2021). CCNB2 participates in the G2/M phase transformation in the eukaryotic cell cycle by activating CDKs (Raina et al., 2021). The research of Meyer Da showed that the up-regulation of protein kinase CDK5 is the main reason for neuronal damage during CIS. Restraining abnormal CDK5 during CIS can protect dopamine neurotransmission, and obstruct excitatory toxicity (Meyer et al., 2014). Meanwhile, extensive studies have shown that overexpression of CCNB2 is associated with the development and deterioration of colorectal, lung, and breast cancers (Ma et al., 2021; Moradpoor et al., 2021; Wang et al., 2021; Toolabi et al., 2022). Consequently, CCNB2 can be used as a potential introduction to study the common pathways of the two diseases.

To describe the diversity of cell types in body or disease development and to reveal the functions and mechanisms of various types of cells, the gene expression differences between individual cells need to be explored. Single-cell transcriptome sequencing (scRNA-seq) technology analyzes transcriptome sequencing data at the single-cell level through unbiased, high-throughput, and high-resolution methodology (Ranzoni et al., 2021). Presently, the scRNA-seq method is extensively used in the fields of tumor, immunity, neural development, and animal genetics, among others (Fan et al., 2020). Therefore, scRNA-seq is an effective tool for mining molecular mechanisms.

In summary, the objective of this study is to evaluate the expression and clinicopathological significance of CCNB2 in CIS and LC using high-throughput datasets from public databases and to know preliminarily about the biological mechanism of CCNB2 in CIS through molecular classification and scRNA-seq analyzes.

## Materials and Methods

### Differential Expression of Cyclin B2 Between Cerebral Ischemic Stroke and Lung Cancer Samples Compared With the Control Group

Two microarray matrixes containing 12 samples from First Affiliated Hospital of Guangxi Medical University [three lung adenocarcinoma (LUAD) vs. three non-LUAD, three lung squamous cell carcinoma (LUSC) vs. three non-LUSC] were used for detecting mRNA expression of CCNB2 (purchased from Fanpu Biotech, Guilin, China) and were included in our study. Afterward, the keywords “CIS” and “lung cancer” were used for data retrieval in the public databases of ArrayExpress^1^, Sequence Read Archive (^2^ SRA), and Gene Expression Omnibus (GEO^3^). The inclusion criteria were as follows: (1) samples containing mRNA expression of CCNB2 in CIS, LC, and control tissues; (2) sample is a homo sapiens; (3) CIS and LC patients did not receive any treatment; and (4) sample is derived from serum, bodily fluids, or tissue of the patient. To illustrate the expression level of CCNB2 in the experimental and control groups as well as the discrimination ability of the samples in the experimental group, the included datasets were evaluated using integration analysis. The standard mean deviation (SMD) and its 95% confidence interval were calculated by extracting the mean ± SD value in each study. In addition, I^2^ > 50% and *p* < 0.05 indicate that the study cohort has significant heterogeneity and thus the random-effect model should be used; otherwise, the fixed model should be used. A test of publication bias was conducted to measure the heterogeneity of the cohort. *p* > 0.05 indicates no significant publication bias in the study cohort. Subsequently, a diagnostic accuracy analysis was conducted to judge the ability of CCNB2 to distinguish the experimental group from the control group, and the best cut-off value was determined through sensitivity (SEN) and specificity (SPEC). Furthermore, True Positive (TP), False Positive (FP), False Negative (FN), and True Negative (TN) were obtained to summarize the results of diagnostic analysis and calculate the SEN, SPEC, negative likelihood ratio (NLR), positive likelihood ratio (PLR), and diagnostic odds ratio (DOR). Finally, the summary receiver operating characteristic (SROC) curve was drawn to obtain the area under the curve (AUC) of SROC according to previous reports (Gao et al., 2021; He et al., 2021; Huang W. Y. et al., 2021; Liang et al., 2021a,b).

### Expression of the Cyclin B2 Protein in Lung Tissue Compared With Lung Adenocarcinoma and Lung Squamous Cell Carcinoma

From the Human Protein Atlas (HPA) database (Colwill and Gräslund, 2011)^4^, the data of the CCNB2 protein expression in lung and LUAD as well as LUSC tissues were obtained. The detailed IHC procedure was conducted as described in the original reference (Colwill and Gräslund, 2011). The expression level was evaluated according to the intensity and density of immunohistochemical staining. For the scores of staining intensities, “Not detected” was 0 points, “Low intensity” was 1 point, “Medium intensity” was 2 points, and “High intensity” was 3 points. For the scores of staining densities, “Negative density” was 0 points, “Weak density” was 1 point, “Moderate density” was 2 points, and “Strong density” was 3 points. To obtain the aggregate score, the intensity and density scores were multiplied to divide the protein expression into 0–9 points.

### Protein-Protein Interaction Network and Enrichment Analysis of Intersected Genes

To identify the potential interaction relationships between genes from the protein level, a PPI network was formed based on 123 intersection genes using the Search Tool for the Retrieval of Interacting Genes (STRING). In addition, according to the KEGG result, another PPI network was also constructed by STRING. To further explore the hub genes with the most potential among the selected genes, Cytoscape v3.7.2 was used to screen the hub genes. To further excavate the potential functions and related molecular mechanisms of the intersection of differentially expressed genes (DEGs) in LUAD, lung squamous cell carcinoma (LUSC), small cell lung cancer (SCLC), and CIS, we entered the intersection genes into R (Version 4.0.4) to conduct gene ontology (GO) and Kyoto Encyclopedia of Genes and Genomes (KEGG) enrichment analysis. GO terms and the KEGG signal pathway with *p* < 0.05 and FDR < 0.1 were identified. Similarly, we used R (Version 4.0.4) to search for the significantly enriched pathways from the Reactome database, and the pathways were selected when *p* < 0.05.

### Regulatory Network of Cyclin B2 in Cerebral Ischemic Stroke

Pearson correlation analysis was conducted to analyze CNNB2 and other gene expressions in the CIS patient cohort. The top 2,000 significant genes were defined as the related genes of CCNB2. Gene co-expression network analysis can effectively identify functional co-expression gene modules related to complex human diseases. Multiscale embedded gene co-expression network analysis (MEGENA) adopts the network embedding paradigm in the topology field. Moreover, gene expression data contain information about the relationships between genes. Consistency clustering is an unsupervised clustering analysis that can identify interrelated genes and verify the rationality of clustering through a resampling-based method. It has a potentially high return in the prognostic analysis and treatment of cancer. Afterward, the R package ConsensusClusterPlus was adopted for the classification of clustering to obtain the K value with the best stability.

### Mutation Analysis of Cyclin B2 in Lung Cancer and Clinical Parameter Analysis

A cell may encounter more than 700,000 DNA damages every day. If these damages cannot be repaired or are incorrectly repaired, they may lead to DNA nucleotide substitution, including single nucleotide variation (SNV), small or large insertions and deletions (indels), copy number variation (CNV), and chromosome rearrangement (GCR). Moreover, DNA replication, transcription, and recombination will destroy DNA stability and further increase the mutation load of the genome. Therefore, somatic mutations can reflect individual environmental exposure and the DNA repair process. R package TCGAbiolinks (Version 2.22.1) was used to download the mutation information of LC patients, and Mutect2 was used to analyze the mutation of CCNB2 in LC. In addition, datasets containing the pathological grade, age, and survival rate of patients were searched to analyze the clinical significance of the CCNB2 mRNA expression.

### Single-Cell Transcriptome Sequencing (scRNA-seq) Analysis of Cyclin B2 in Brain Organoids and Lung Adenocarcinoma

The GSE184409 and GSE189357 dataset of GEO contains six brain organoids and nine LUAD tissues for dissecting the cells’ transcriptional traits. The subpopulations of cells were obtained through linear dimensionality reduction principal component analysis (PCA) and uniform manifold approximation and projection (UMAP) clustering. The marker genes and disease difference molecules in the cell subpopulations were analyzed and screened using the Wilcox rank-sum test. The marker genes of each population obtained in this dataset were compared with the marker molecules collected in the articles. According to the relative expression of the single-cell genes after integrating and correcting the batch effect, the differentially expressed gene set was selected as the variable to conduct the trajectory construction function and construct the single-cell development trajectory diagram in Monocle 2 software package, which was displayed with 2D visual results.

### Statistical Analysis

In this study, alongside the statistical analysis explained above, when the CCNB2 expression presented a normal distribution, R 4.0.3 was used for Student’s *t*-test. If the CCNB2 expression was abnormally distributed, a non-parametric test was used to compare the differential expression of CCNB3 in the experimental and control groups. GraphPad Prism 8.0 was employed to draw a box chart showing the distribution of the CCNB2 expression in the experimental and control groups. The AUC was used to evaluate the ability of the CCNB2 expression to distinguish between the experimental and control groups. STATA 14.0 was employed to calculate the SMD and 95% confidence interval, systematically reflecting the expression of CCNB2. sROC was calculated to illustrate the ability of CCNB2 to distinguish the experimental group from the control group. The Kaplan-Meier plotter was used to analyze the effect of the CCNB2 mRNA expression on the survival rate of patients. Using the median value, patients were divided into two groups according to the high and low expressions of the CCNB2 mRNA, a Kaplan-Meier curve was drawn, and the hazard ratio (HR) was calculated to judge the predictive ability of the CCNB2 mRNA expression on the patients’ survival rate.

## Results

### Evidence From Multiple High-Throughput Data Suggests That Cyclin B2 Is Significantly Up-Expressed in the Cerebral Ischemic Stroke and Lung Cancer Samples

**FIGURE 1:**
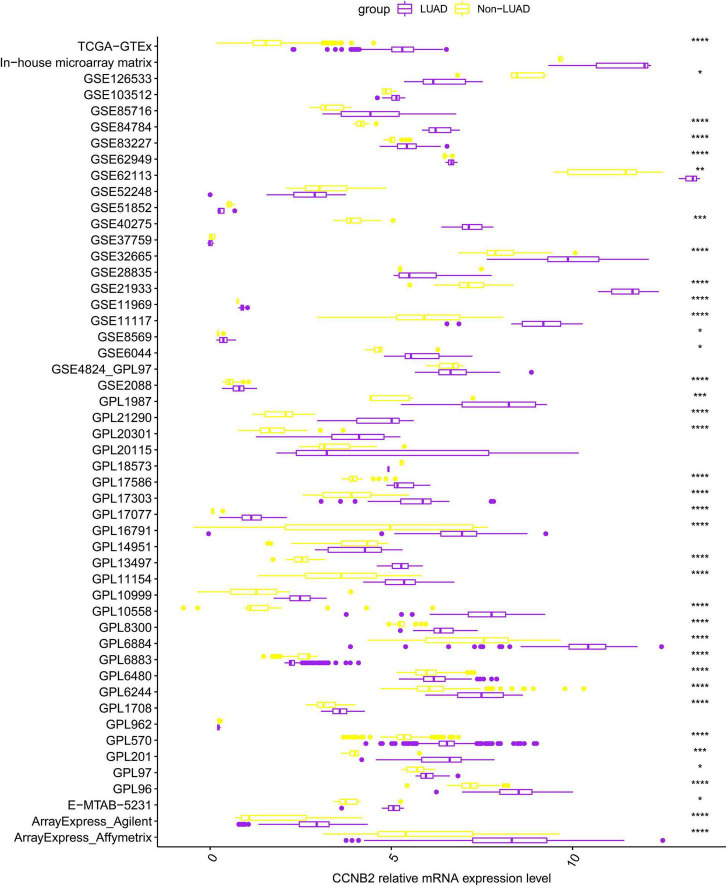
Differential expression of cyclin B2 (CCNB2) between lung adenocarcinoma (LUAD) and non-LUAD samples. ****P < 0.0001; ***P < 0.001; **P < 0.01; *P < 0.05.

**FIGURE 2:**
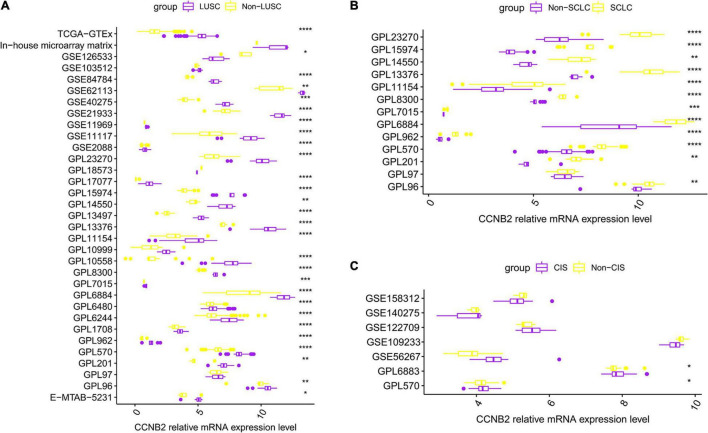
Differential expression of cyclin B2 (CCNB2) between cerebral ischemic stroke (CIS) and lung cancer (LC) samples compared with control group. **(A)** Box-plot of each dataset included in expression analysis of CCNB2 in lung squamous cell carcinoma (LUSC). **(B)** Box-plot of each dataset included in expression analysis of CCNB2 in small-cell lung carcinoma (SCLC). **(C)** Box-plot of each dataset included in expression analysis of CCNB2 in CIS. ****P < 0.0001; ***P < 0.001; **P < 0.01; *P < 0.05.

**FIGURE 3:**
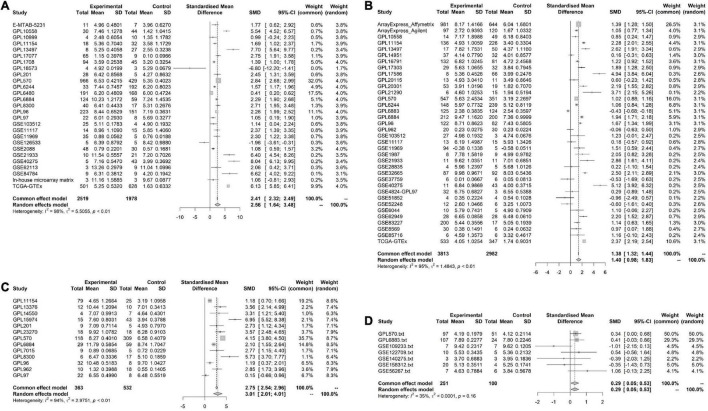
Pooled standard mean deviation (SMD) forest plot for CNNB2 between cerebral ischemic stroke (CIS) and lung cancer (LC) samples compared with control group. **(A)** standard mean deviation (SMD) forest plot for CNNB2 in lung squamous cell carcinoma (LUSC). **(B)** SMD forest plot for CNNB2 in lung adenocarcinoma (LUAD). **(C)** SMD forest plot for CNNB2 in small cell lung cancer (SCLC). **(D)** SMD forest plot for CNNB2 in CIS.

**FIGURE 4:**
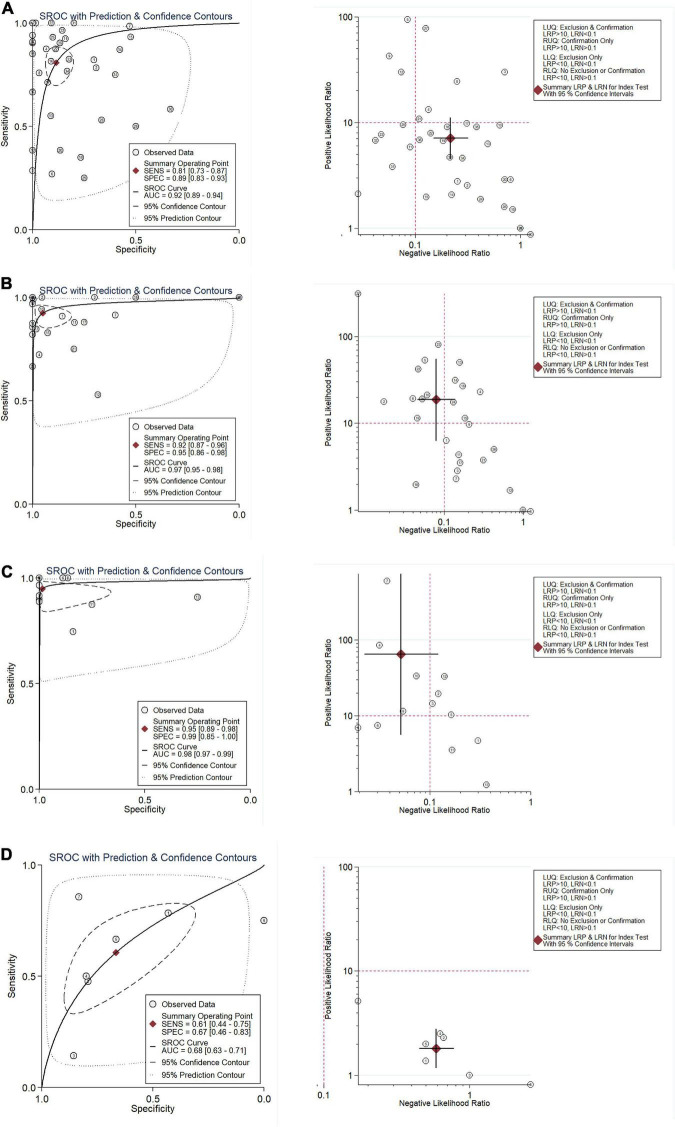
Integration analysis of CNNB2 in stroke, lung cancer (LC) and control samples for in-house and external datasets. **(A)** Summarized receiver operating characteristic (sROC) curve and likelihood ratio of cyclin B2 (CCNB2) reflected ability of CCNB2 expression in distinguishing experimental samples from control samples in lung adenocarcinoma (LUAD). **(B)** Summarized receiver operating characteristic (sROC) curve and likelihood ratio of CCNB2 reflected ability of CCNB2 expression in distinguishing experimental samples from control samples in LUSC. **(C)** Summarized receiver operating characteristic (sROC) curve and likelihood ratio of CCNB2 reflected ability of CCNB2 expression in distinguishing experimental samples from control samples in small cell lung cancer (SCLC). **(D)** Summarized receiver operating characteristic (sROC) curve and likelihood ratio of CCNB2 reflected ability of CCNB2 expression in distinguishing experimental samples from control samples in cerebral ischemic stroke (CIS).

Overall, 103 high-throughput datasets related to the study, including the CCNB2 expression, were bring into (Figures 1, 2 and Supplementary Table 1) while it failed to collect tissue samples of CIS patient and bodily fluids samples of LC patient. Each dataset was strictly under the corresponding CIS or LC diagnosis guidelines, and the experimental design of each included study was approved by the relevant review committee. In the included datasets, *P* < 0.05 indicated great heterogeneity, and thus the random-effect model was used. Integration analysis indicated that CCNB2 was up-regulated in the CIS and LC samples (Figure 3). Moreover, the funnel plot was distributed symmetrically, which indicated no significant publication bias in our study (Supplementary Figure 1A). Based on calculation of SEN and SPEC (Supplementary Figures 2, 3), the results of the SROC discriminative accuracy and the likelihood ratio reflected the fine identification ability of CCNB2 in CIS and LC (Figure 4 and Supplementary Figure 1B).

### Verification of Upregulation of Cyclin B2 Protein Expression Level in Lung Adenocarcinoma and Lung Squamous Cell Carcinoma

**FIGURE 5:**
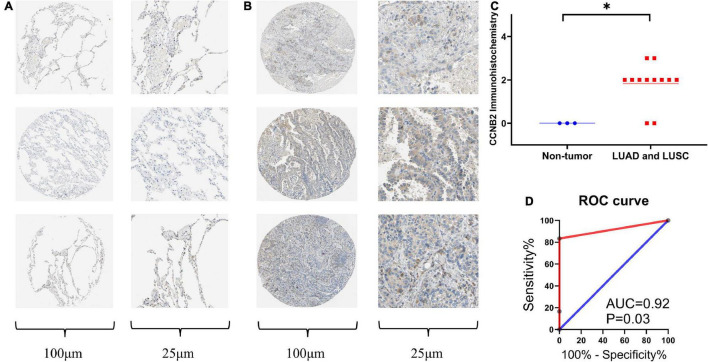
Immunohistochemistry (IHC) staining of cyclin B2 (CCNB2) in lung adenocarcinoma (LUAD) and non-tumor tissues (Antibody CAB009575). **(A)** Negative IHC staining of CCNB2 in non-tumor tissues; **(B)** Positive IHC staining of CCNB2 in LUAD and LUSC tissues; **(C)** Scatter diagram of IHC staining displayed expression analysis of CCNB2; **(D)** The receiver operator characteristic (ROC) curve of IHC staining displayed expression analysis of CCNB2. *P < 0.05.

Immunohistochemistry (IHC) was conducted to detect the CCNB2 expression in 12 LUAD and LUSC tissues as well as three non-tumor lung tissues. The representative pictures of IHC staining are shown in Figure 5A. CCNB2 was found to be negatively expressed in the lung tissues. Significantly, CCNB2 was expressed in partial LUAD and LUSC cells (Figure 5B). After analyzing 15 samples, we discovered that the expression intensity of CCNB2 in the LUAD and LUSC tissues was significantly higher than that in the non-tumor lung tissues (Figure 5C, *p* < 0.05). Based on the cut-off value of the immunohistochemical score, we could divide the samples into two groups, with AUC = 0.92 (Figure 5D).

### Protein-Protein Interaction Network and Enrichment Analysis Suggested Key Status of Cyclin B2 in Cerebral Ischemic Stroke and Lung Cancer

**FIGURE 6:**
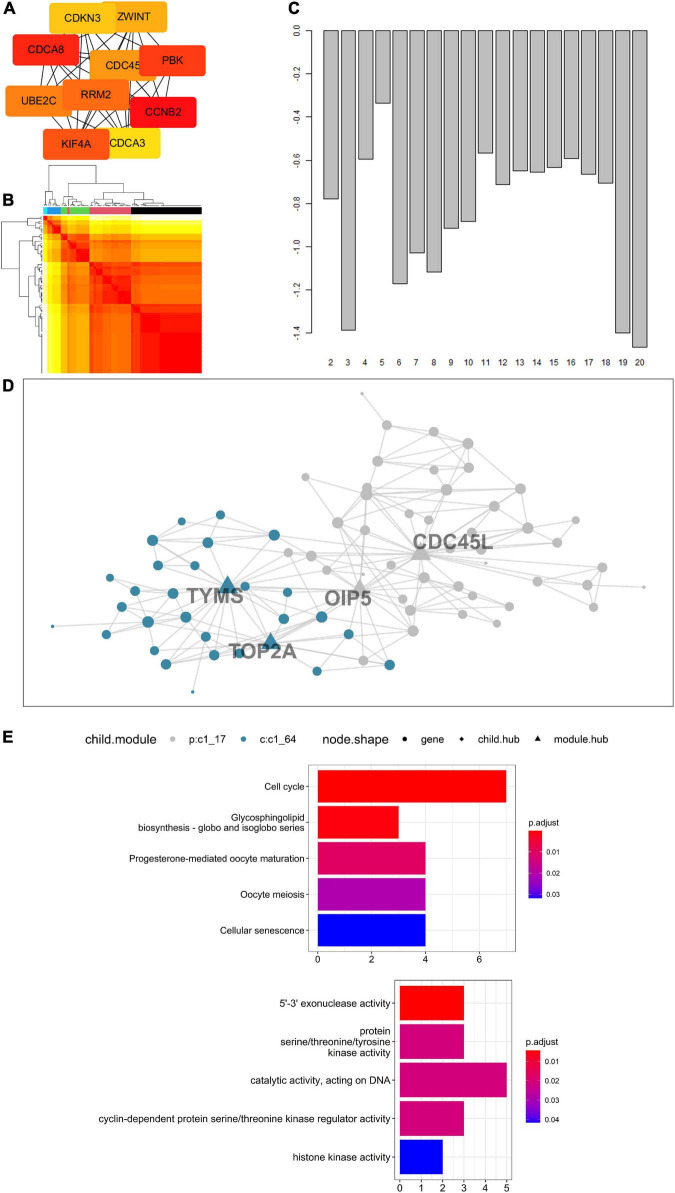
Enrichment analysis of genes up-regulated on cerebral ischemic stroke (CIS) and lung cancer (LC) and Multiscale Embedded Gene Co-expression Network Analysis (MEGENA) of cyclin B2 (CCNB2) in CIS cohort. **(A)** The hub genes of intersected differentially expressed genes (DEGs). **(B)** Heatmap of scale cluster. **(C)** Result of mean rank using majority vote algorithm. **(D)** Network diagram of CCNB2 module genes. **(E)** Kyoto Encyclopedia of Genes and Genomes (KEGG) and Gene Ontology (GO) enrichment analysis for module genes co expressed with CCNB2.

Concerning 123 intersected DEGs screened from LUAD, LUSC, SCLC, and CIS, a PPI network was built to express the connectedness and generality among the DEGs. Ten molecules with strong degree bounds were identified as central genes, including CCNB2, cell division cycle associated 8 (CDCA8), PDZ-binding kinase (PBK), kinesin family member 4A (KIF4A), ribonucleotide reductase regulatory subunit M2 (RRM2), ubiquitin-conjugating enzyme E2 C (UBE2C), cell division cycle 45 (CDC45), ZW10 interacting kinetochore protein (ZWINT), cyclin-dependent kinase inhibitor 3 (CDKN3), and cell division cycle associated 3 (CDCA3) (Figure 6A). To further explore the signal pathways of these genes, a functional and pathway enrichment analysis of the 123 genes was conducted. According to the result, these genes were significantly enriched in the cell cycle, regulation of the cell cycle process, and the cell cycle checkpoints, among others (The top three terms, Supplementary Figure 4), which indicates CCNB2 exerts a significant role in the gene set.

### Biological Functions of the Cyclin B2 Module Genes in Cerebral Ischemic Stroke

The CCNB2-related genes obtained by correlation analysis were expressed in a co-expression network (Figures 6B,C), and a module comprising 76 genes was obtained (Figure 6D). The hub gene included thymidylate synthetase (TYMS), DNA topoisomerase II alpha (TOP2A), Opa-interacting protein 5 (OIP5), and cell division cycle 45-like (CDC45L). Functional enrichment analysis indicates that these genes are mainly enriched in 5’-3’ exonuclease activity, protein serine/threonine/tyrosine kinase activity, catalytic activity acting on DNA, and cyclin-dependent protein serine/threonine kinase regulator activity, and histone kinase activity (Figure 6E). Pathway analyzes of module genes were enriched in the cell cycle, oocyte meiosis, and p53 signaling pathway (Figure 6E).

### Tumor Mutation Characteristics and Clinical Significance of Cyclin B2

**FIGURE 7:**
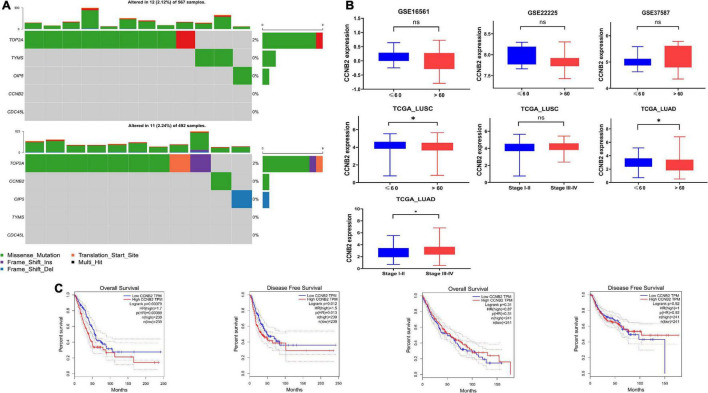
Mutation analysis and clinical significance of cyclin B2 (CCNB2). **(A)** Mutation analysis of CCNB2 and hub genes of CCNB2 module in lung cancer (LC). **(B)** The clinic-pathological significance of CCNB2 in LC and cerebral ischemic stroke (CIS). **(C)** Kaplan-Meier survival curves for overall survival and progression-free survival (PFS) of TCGA-LUSC and TCGA-LUAD grouped by expression of CCNB2. *P < 0.05.

The main important mutation type of CCNB2 module genes is a missense mutation (Figure 7A). Among the module genes, TOP2A was found to have the highest mutation frequency, including missense mutation, frameshift insertion mutation, and multihit, in LUAD and LUSC. More importantly, the expression of CCNB2 was related to age, pathological stage, overall survival (OS), and progression-free survival (PFS) of Euro-American LUAD patients (Figures 7B,C).

### Genotyping Based on Cyclin B2 Co-Expressed Genes Has a Significant Pathway Enrichment Difference

**FIGURE 8:**
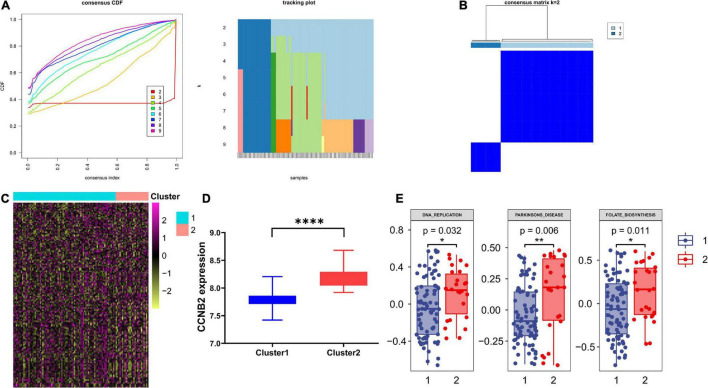
Consensus clustering analysis of co expression genes of cyclin B2 (CCNB2) in cerebral ischemic stroke (CIS). **(A)** Line chart and heat map pointed out *k* = 2. **(B)** Heatmap of clustering effect while *k* = 2. **(C)** Gene set variation analysis (GSVA) of 2 clusters. **(D)** 2 clusters showed significant differentially expressed CCNB2 expression **(E)** 2 clusters showed significant pathway enrichment differences. ****P < 0.0001; **P < 0.01; *P < 0.05.

Based on the consensus clustering method, the CIS samples were divided into two subgroups (Figures 8A,B). The Student’s *t*-test indicated a distinct discrepancy in the distribution of CCNB2 (Figure 8D) and pathway enrichment (Figure 8C) in two clusters. It was demonstrated that the significant specialties of clusters with higher CCNB2 expression are higher activity folate biosynthesis, basal transcription factors, and Parkinson’s disease (PD) pathway (Figure 8E).

### Single-Cell Transcriptome Sequencing (scRNA-seq) Analysis Suggests That Cyclin B2 Is Up-Regulated on GABAergic Neurons in Brain Organoids and T Cells in Lung Adenocarcinoma

**FIGURE 9:**
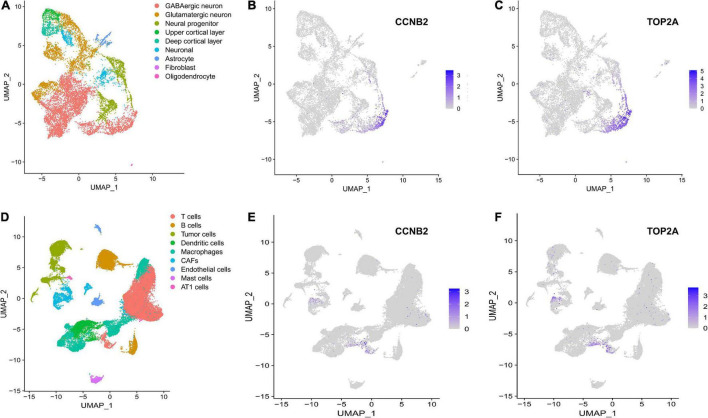
scRNA-seq analysis process of GSE184409 and GSE189357. **(A)** Uniform manifold approximation and projection (UMAP) distribution of 9 annotated cell types in brain organoids. **(B)** Distribution of cyclin B2 (CCNB2) expression in cell clusters of brain organoids. **(C)** Distribution of DNA topoisomerase II alpha (TOP2A) expression in cell clusters of brain organoids. **(D)** UMAP distribution of 9 annotated cell types in lung adenocarcinoma (LUAD). **(E)** Distribution of CCNB2 expression in cell clusters of LUAD. **(F)** Distribution of TOP2A expression in cell clusters of LUAD.

**FIGURE 10:**
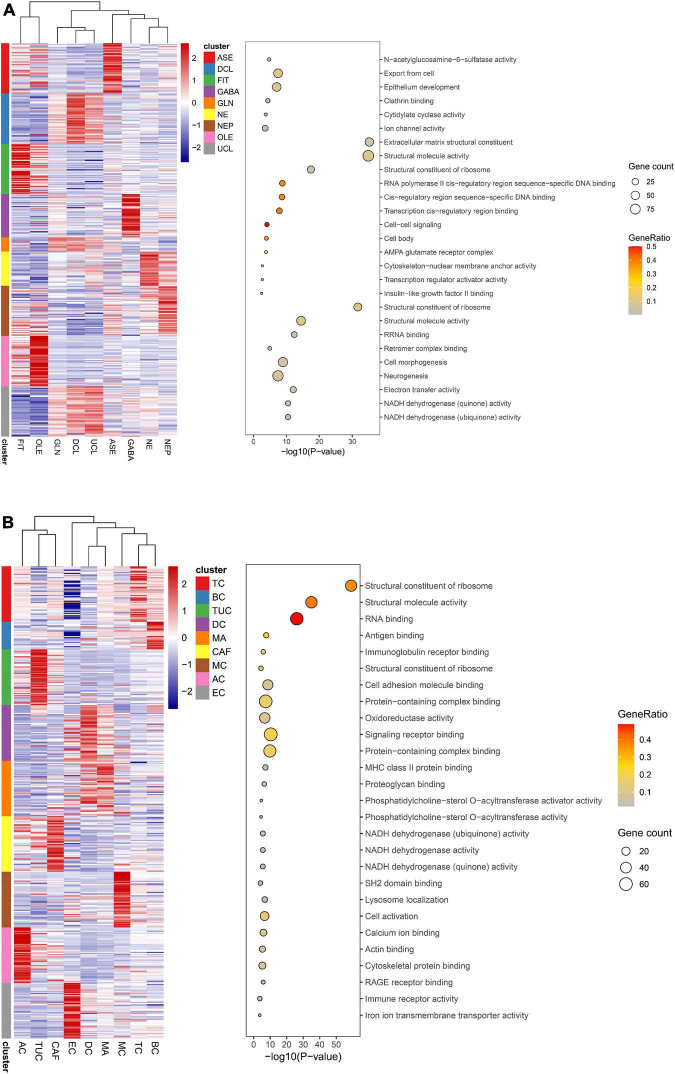
Function analysis of cell types. **(A)** Function analysis of 9 annotated cell types in brain organoids. **(B)** Function analysis of 9 annotated cell types in lung adenocarcinoma (LUAD).

**FIGURE 11:**
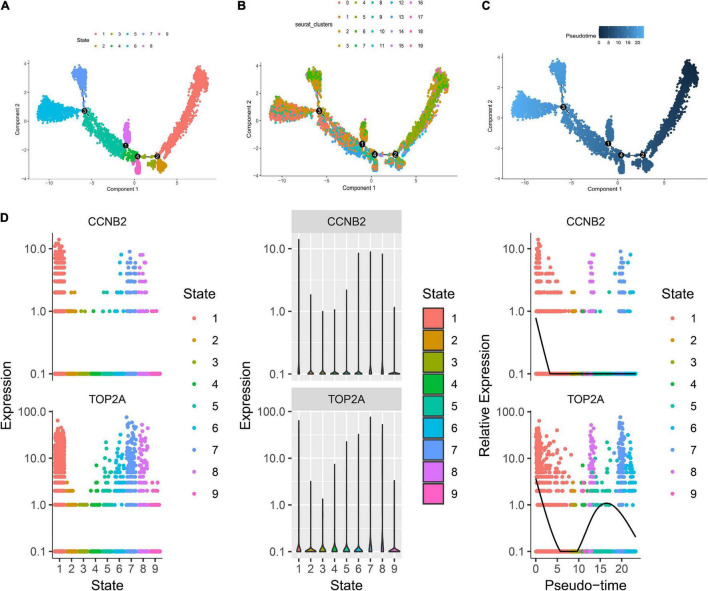
Trajectory analysis of brain organoids cell. **(A)** The two-dimensional distribution map of state shows the trajectory results of brain organoids cells. **(B)** Display diagram of cell development timing score calculated according to development trajectory analysis. **(C)** The two-dimensional trajectory distribution map of cluster. **(D)** The dynamic changes of the expression level of cyclin B2 (CCNB2) and DNA topoisomerase II alpha (TOP2A) during development.

**FIGURE 12:**
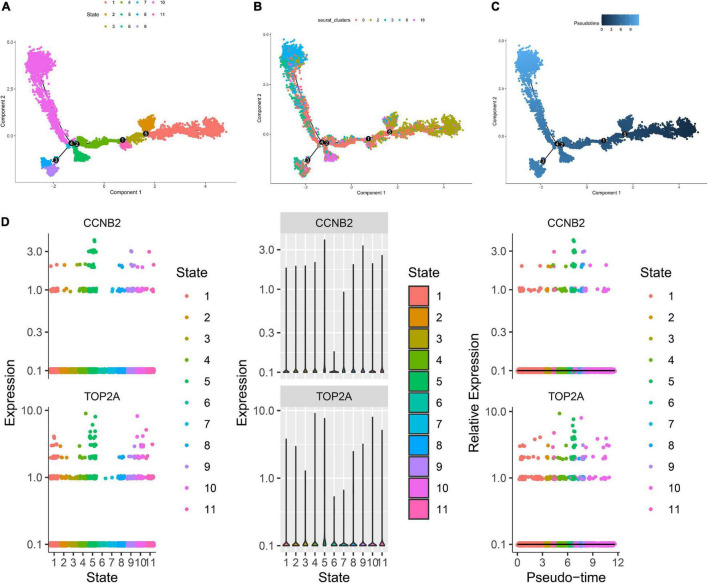
Trajectory analysis of T cells in lung adenocarcinoma (LUAD). **(A)** The two-dimensional distribution map of state shows the trajectory results of T cells. **(B)** Display diagram of cell development timing score calculated according to development trajectory analysis. **(C)** The two-dimensional trajectory distribution map of cluster. **(D)** The dynamic changes of the expression level of cyclin B2 (CCNB2) and DNA topoisomerase II alpha (TOP2A) during development.

The harmony function was adopted to rectify the sample heterogeneity of the expression matrix. The cells were identified as different clusters using an unsupervised clustering method, and the up-regulated molecules of the cell subpopulation were gained through differential screening. The up-regulated genes compared markers collected in the CellMarker database^5^ and marker molecules collected in the articles to select markers for the study. Overall, the cells subpopulation was marked as cells (Figures 9A,D) in brain organoids and LUAD (Brain organoids: GABAergic neurons, glutamatergic neuron, neural progenitor, upper cortical layer, deep cortical layer, neuronal, astrocyte, fibroblast, and oligodendrocyte; LUAD: T cells, B cells, tumor cells, dendritic cells, macrophages, cancer-associated fibroblasts (CAFs), endothelial cells, mast cells, and AT1 cells) indicating the involvement of different biological processes (Figure 10). CCNB2 and TOP2A were significantly over-expressed in GABAergic neurons (Figures 9B,C) which suggested that CCNB2 was related to repair after CIS damage. Simultaneously, CCNB2 was found up-regulated on T cells expressing proliferative molecules including TOP2A, marker of proliferation Ki-67 (MKI67), and proliferating cell nuclear antigen (PCNA) in LUAD (Figures 9E,F and Supplementary Figure 5). DEGs among the cell groups were taken as the reference gene set, then a pseudo-temporal algorithm was employed to predict the dynamic development trajectory of the cells (Figures 11, 12).

## Discussion

This study indicated the up-regulation of the CCNB2 mRNA expression in CIS and LC through multilevel evidence. Simultaneously, by analyzing the CCNB2 co-expression genes and scRNA-seq data, the potential biological pathways CCNB2 might involve in were explored, which provides a new perspective for researching the mechanisms of the shared biological pathways between CIS and LC. Our study has the following advantages. First, the study included 103 high-throughput datasets, including 6946 experimental and 5592 control samples, which strongly confirmed the up-regulation of CCNB2 mRNA in CIS and LC through integrated analysis. Additionally, it is of great significance to study LC using tissue samples, because the oncogenesis of LC originates from cytology carcinogenesis. Second, it is the first systematic study of CCNB2 considering the shared biological function between CIS and LC, thus enriching the theoretical content of cyclin family genes. Last, there is still a lack of genome-based and high-throughput data analysis methods to mine the biological mechanism of CCNB2. More importantly, the expression pattern of CCNB2 at the single-cell level in brain organoids and LUAD was explored. Overall, this study comprehensively analyzed the potential biological mechanism of CCNB2 in CIS and LC, which indicated that CCNB2 promotes LC and CIS by regulating the cell cycle, protein kinase activity, and interacting with TOP2A.

Initially, through PPI analysis of crossed high-expression genes in CIS and LC, we found that CCNB2 may be an important gene in the common pathway. Furthermore, using MEGENA analysis, a gene module of CIS including 76 genes was obtained. The function and pathway enrichment analysis of the CCNB2 module genes showed that CCNB2 may participate in the formation of CIS and tissue damage caused by CIS, such as “cell cycle,” “protein kinase activity,” and “glycosphingolipid biosynthesis” through the module gene network. Importantly, previous studies suggested that the change in protein kinase activity was related to the occurrence of CIS (Riebeling et al., 2021). Receptor interacting serine/threonine kinase 3 (RIPK3) was an important member of the serine/threonine protein kinase family and a key regulator of programmed necrosis (Liu et al., 2021). It was found that in the advanced stage of atherosclerosis, about half of the death of macrophages in the plaque was caused by programmed necrosis regulated by RIPK (Zhang et al., 2021). Meanwhile, the expression of RIPK3 in carotid atherosclerotic plaque with necrotic part was significantly higher than that in other tissues (Tian et al., 2016). Mitogen-activated protein kinase activated protein kinase 2 (MK2) was mainly activated by mitogen-activated protein kinase 14 phosphorylation (MPK14) (Zhao et al., 2021). It was also found *in vivo* experiments that hypercholesterolemic low density lipoprotein receptor (IDLR) (-/-)/MK2(-/-) mice could resist atherosclerosis with reduced accumulation of lipids and macrophages in the aorta (Jagavelu et al., 2007). Meanwhile, the study of Ebrahimian T showed that the activation of MK2 mediates the production of inflammatory mediators and increased blood pressure regulated by angiotensin (Ebrahimian et al., 2011). Simultaneously, MAPKs family played an important role in the process of neuronal apoptosis by participating in the regulation of apoptosis related-genes (Zhou et al., 2021). Whether it was transient or permanent ischemic injury, MAPKs expression level altered significantly, and inhibiting its cascade reaction could improve the cerebral ischemic injury in animal models (Yin et al., 2021). Therefore, the regulation of mitogen-activated protein kinase pathway may be an effective way to inhibit cerebral ischemic neuronal injury and protect neural function. The cell cycle is divided into the G1, S, G2, and M phases, and the development of cells depends on the normal operation of the cell cycle, which is an internal component of cell differentiation and proliferation (Lezaja and Altmeyer, 2021). The cell cycle is mainly regulated by cyclins and CDKs. Cyclins are abnormally expressed in CIS, while CDKs play an important role in CIS injury (Saraireh et al., 2012). Inhibiting the activity of the cell cycle has a significant neuroprotective effect. Trimsit et al. found that the expression of CyclinD1 and CDK4 in the penumbra, after cerebral ischemia, is a key marker to evaluate whether ischemic neurons enter the cell cycle again (Wood and Endicott, 2018). Additionally, Katchanov et al. found that in the rat model of cerebral ischemia-reperfusion, the deletion of endogenous CDKI p16INK4a can lead to the delayed death of striatal neurons (Katchanov et al., 2001), indicating that there is a relationship between the cell cycle and neuronal injury. Concurrently, many studies have shown that the composition and metabolism of glycosphingolipids on the surface of the tumor cell membrane will undergo changes, such as the emergence of new glycosphingolipid antigens and the transformation of glycosphingolipid structure, among others (Yu et al., 2020). The alternation correlates with the occurrence and development of tumors and can be distinguished as a tumor marker (Moghimi et al., 2021). Furthermore, inhibiting the high expression of related glycosphingolipids will have a clear palliative effect on the development of tumors and will help to block the metastatic pathway of tumors. For example, Salk found that the sialylation level of the glycosphingolipid sugar chain of cells increased with high malignancy or high metastatic potential, indicating that sialic acid glycosphingolipid may promote the metastasis and deterioration of tumor cells (Yang et al., 2021). These studies have suggested a potential association between cell cycle activity and the related metabolism between LC and CIS.

Subsequently, TOP2A as a hub gene of the CCNB2 co-expression module in CIS was found to have the highest mutation frequency, including missense mutation, frameshift insertion mutation, and multihit, in LUAD and LUSC. DNA topoisomerase is the general name of enzymes that can catalyze the conversion of DNA topoisomerase (Crewe and Madabhushi, 2021). TOP2A widely exists in eukaryotic and prokaryotic cells and mainly regulates DNA topology by participating in DNA division, repair, recombination, replication, and transcription (Di Felice and Camilloni, 2021). Among them, TOP2A is mainly distributed in the nucleus and participates in the cell cycle pathway, which is closely related to cell proliferation and apoptosis (Tian et al., 2021). By analyzing the effect of the expression level of TOP2A protein on the proliferation and invasion of tumor cells, the researchers found that the expression of TOP2A protein was closely related to the proliferation and invasion of LC cells (Kanne et al., 2021). However, there are few studies on TOP2A in the nervous system. Recently, Thakurela found that small molecule inhibitors of TOP2 can destroy the formation of embryonic stem cells and lead to the down-regulation of genes of embryonic pluripotency (Thakurela et al., 2013). Studies by Lauren F Harkin have shown that TOP2A is involved in regulating the acquisition and maintenance of activated astrocyte stem cell characteristics, which may be mediated by transcription factor Paired Box 6 (PAX6) (Harkin et al., 2016). More importantly, CCNB2 and TOP2A were found specifically over-expressed in GABAergic neurons by scRNA-seq analysis. GABA is an inhibitory neurotransmitter mainly existing in the central nervous system and widely distributed in the brain and spinal cord (Sanchez-Vives et al., 2021). The GABAergic signaling system comprises glutamate decarboxylase (GAD), GABA, GABA transporter, and GABA receptor (GABAR) (Han et al., 2021). In the cerebral cortex, the excitatory signals provided by glutamatergic neurons and the inhibitory signals provided by GABAergic neurons always complement each other to achieve the balance of excitation and inhibition (Bell et al., 2021). Jiang conducted an in-depth study on how the activity of GABAergic neurons affected the rehabilitation of neurological function after stroke (Jiang et al., 2017). The results showed that inhibiting the activity of GABAergic neurons in the striatum could promote the improvement of neurological function and reduce the volume of brain atrophy in post-stroke mice. In addition, these protective effects may be related to the inhibition of GABAergic neuron activity and the promotion of the nutritional factor fibroblast growth factor (FGF) up-regulation in vascular endothelial cells. The above-mentioned studies will help us to further understand the pathological process of CNS injury. Additionally, CCNB2 was found up-regulated on T cells expressing proliferative molecules. Pathway enrichment analysis of upregulated genes on the cluster indicates that CCNB2 promotes LUAD *via* interleukin 18 (IL-18) signaling pathway and vascular endothelial growth factor A (VEGFA)-VEGFR2 signaling pathway. As a proinflammatory cytokine, IL-18 plays an important role in the occurrence and development of infectious diseases and tumors (Gupta A. et al., 2021). Gu RH found that IL-18 exerts a fine prognosis effect on LC patients (Gu et al., 2020). VEGFA is a protein and specific mitotic agent for endothelial cells (Ling et al., 2021). Combined with receptors, VEGFA can promote the division and proliferation of vascular endothelial cells and increase microvascular permeability (Kajal et al., 2021; Lee et al., 2021). Many studies have confirmed that VEGFA is up-regulated in non-small cell lung cancer (NSCLC) tissues and is also significantly positively correlated with lymph node metastasis of NSCLC (Wei et al., 2021; Xia Y. et al., 2021). At the same time, it is also an effective prognostic factor for NSCLC (Huang Y. S. et al., 2021; Shi et al., 2021). In summary, the above results indicate that CCNB2 can be a potential target for treatment strategies for CNS injury repair as well as LC.

Although we attempted to analyze the relationship between LC and CIS using CCNB2 on multiple levels, the following limitations still existed in this study. First, the clinical specimens of patients were not collected, which hindered the collection of clinical parameters, such as tumor, node, and metastasis (TNM) staging. Second, incomplete information prevented us from performing adequate follow-up of patients. There were also insufficient data in the major gene databases and online analysis websites to analyze the influence of CCNB2 expression on the prognosis of CIS and LC. Additionally, the lack of comparison of CCNB2 expression data between LC-related CIS and LC combined with CIS leads to the deficiency in the persuasion of this study. Overall, our results still need to be further confirmed by experiments *in vitro* and vivo.

In summary, multiple inferences were provided for the up-regulation of CCNB2 in LC and CIS. The effect of CCNB2 on the common pathway between LC and CIS may indicate a foreground of CCNB2 as a biomarker and therapeutic target for LC-related CIS.

## Data Availability Statement

The datasets presented in this study can be found in online repositories. ArrayExpress (https://www.ebi.ac.uk/arrayexpress/), Sequence Read Archive (https://trace.ncbi.nlm.nih.gov/Traces/sra/, SRA), and Gene Expression Omnibus (GEO, https://www.ncbi.nlm.nih.gov/geo/).

## Ethics Statement

This study was conducted in accordance with the declaration of Helsinki and approved by the Ethics Committee of The First Affiliated Hospital of Guangxi Medical University (protocol number #2015-KY-Guoji-259), China.

## Author Contributions

M-JL, Z-GH, and GC conceived and designed the present study and revised the manuscript. S-BY analyzed the data and drafted the manuscript. G-SL drafted the manuscript. TW, D-SH, Z-JL, LC, and ZY guided statistical analysis. YY analyzed the data. G-YC, JW, B-TY, R-XX, and L-YL filtered and extracted data. All authors contributed to the article and approved the submitted version.

## Conflict of Interest

The authors declare that the research was conducted in the absence of any commercial or financial relationships that could be construed as a potential conflict of interest.

## Publisher’s Note

All claims expressed in this article are solely those of the authors and do not necessarily represent those of their affiliated organizations, or those of the publisher, the editors and the reviewers. Any product that may be evaluated in this article, or claim that may be made by its manufacturer, is not guaranteed or endorsed by the publisher.

## Footnotes

1
https://www.ebi.ac.uk/arrayexpress/

2
https://trace.ncbi.nlm.nih.gov/Traces/sra/

3
https://www.ncbi.nlm.nih.gov/geo/

4
https://www.proteinatlas.org/

5
http://biocc.hrbmu.edu.cn/CellMarker/index.jsp
