# Supplementary material for: Upregulation of CCNB2 and Its Perspective Mechanisms in Cerebral Ischemic Stroke and All Subtypes of Lung Cancer: A Comprehensive Study
Source: Front Integr Neurosci. 2022 Jul 19;16:854540. doi: 10.3389/fnint.2022.854540 (PMC9344069; doi:10.3389/fnint.2022.854540)
Supplement: Supplementary file 1 [file Data_Sheet_1.docx]

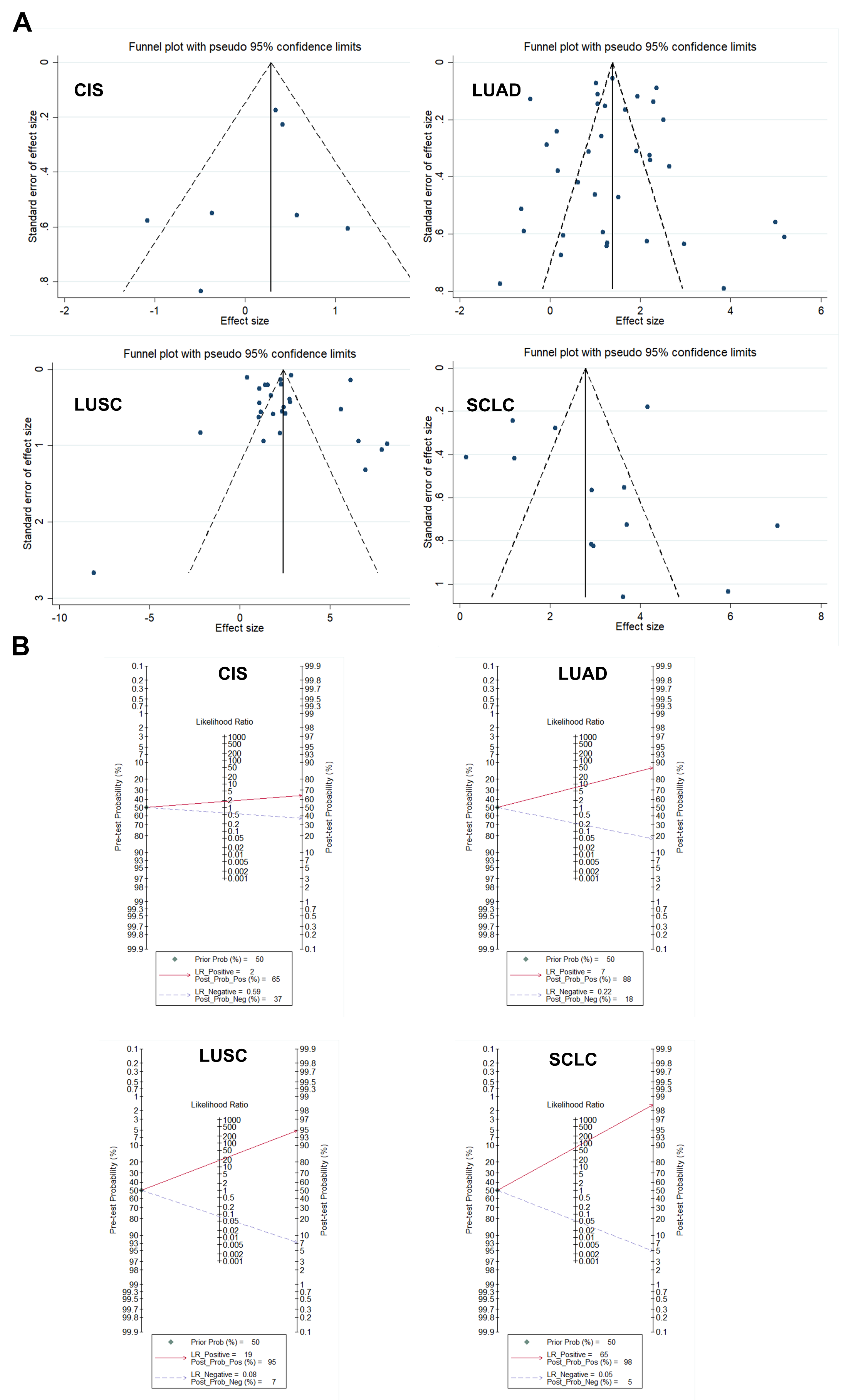


FIGURE S1. Comprehensive analysis of CCNB2 in LC and CIS datasets.

(A) Funnel plot indicating public bias in CIS, LUAD, LUSC, and SCLC.

(B) Fagan plot of CCNB2 in CIS, LUAD, LUSC, and SCLC.

.


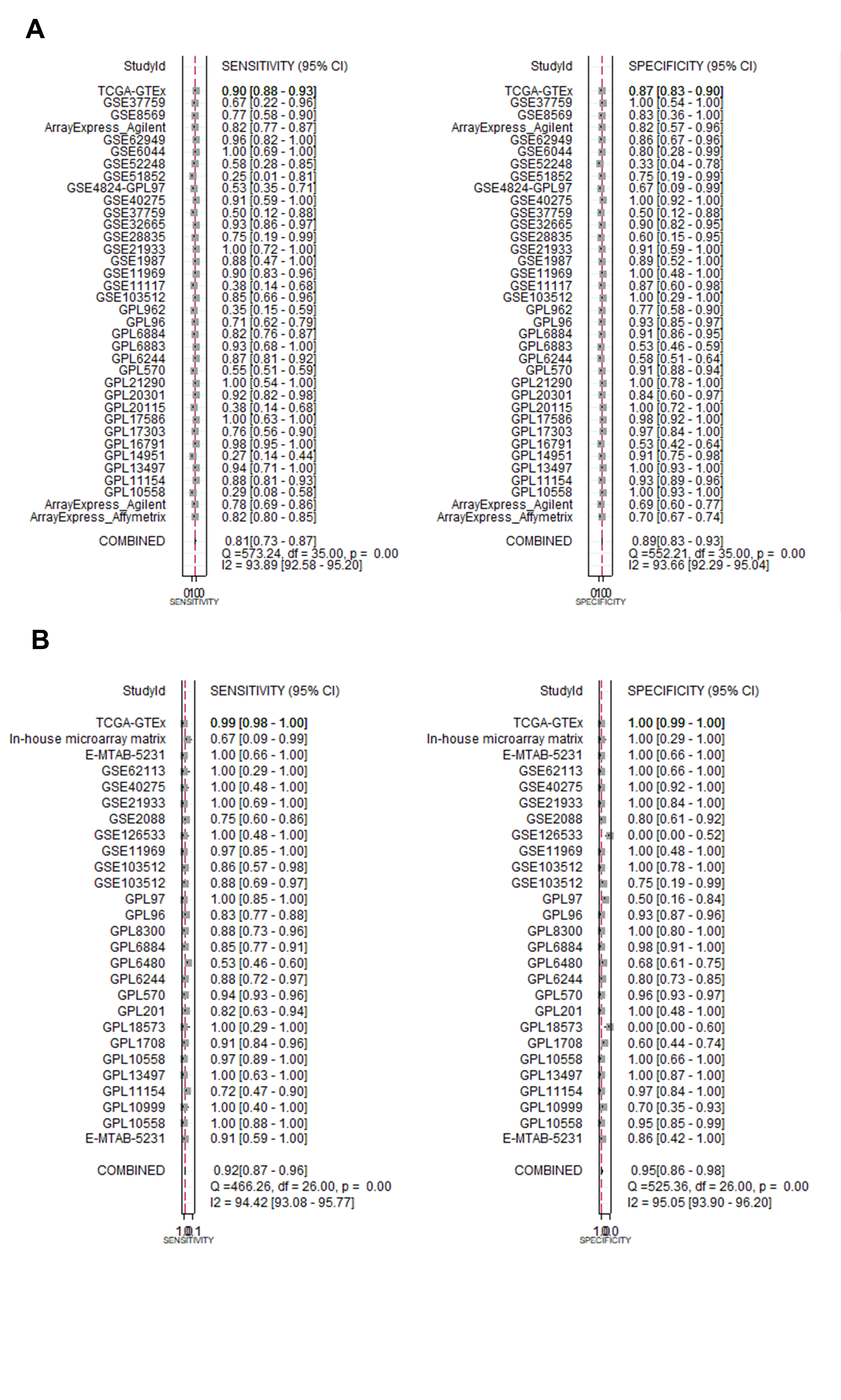


FIGURE S2. Specificity and sensitivity analysis of CCNB2 in LUAD and LUSC.

(A) Forest plot showing specificity and sensitivity of CCNB2 in LUAD.

(B) Forest plot showing specificity and sensitivity of CCNB2 in LUSC.


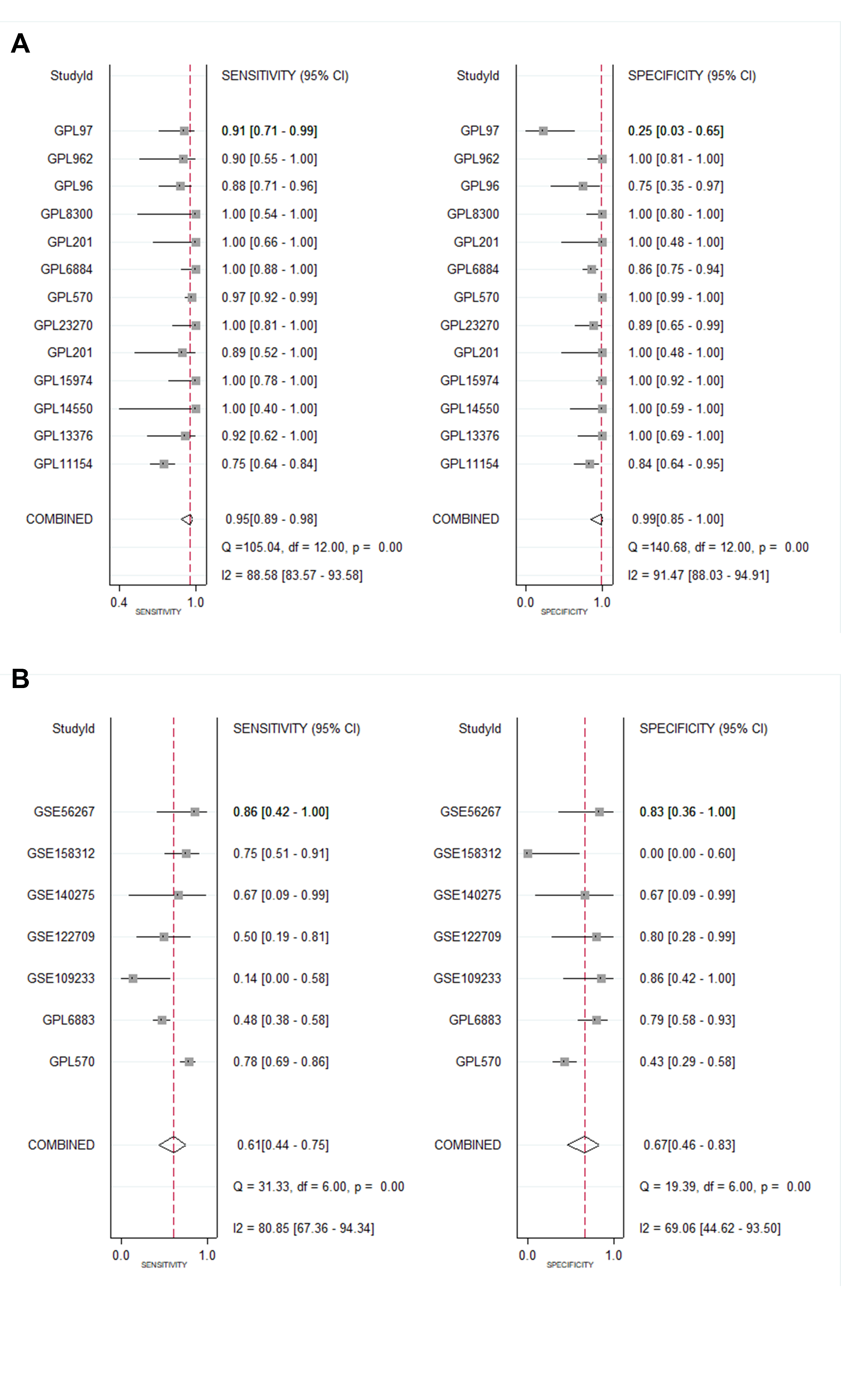


FIGURE S3. Specificity and sensitivity analysis of CCNB2 in SCLC and CIS.

(A) Forest plot showing specificity and sensitivity of CCNB2 in SCLC.

(B) Forest plot showing specificity and sensitivity of CCNB2 in CIS.


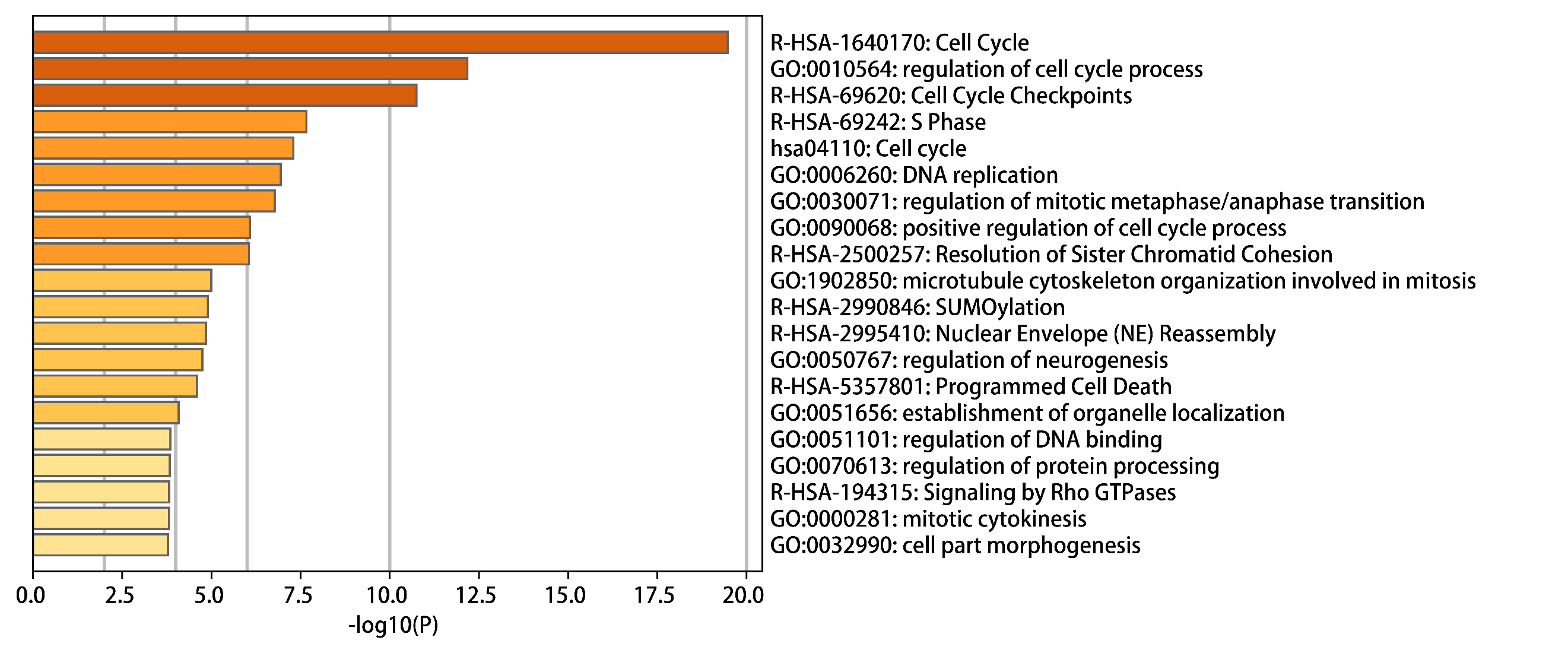


FIGURE S4. Functional enrichment analysis of Intersected DEG genes between LC and CIS.


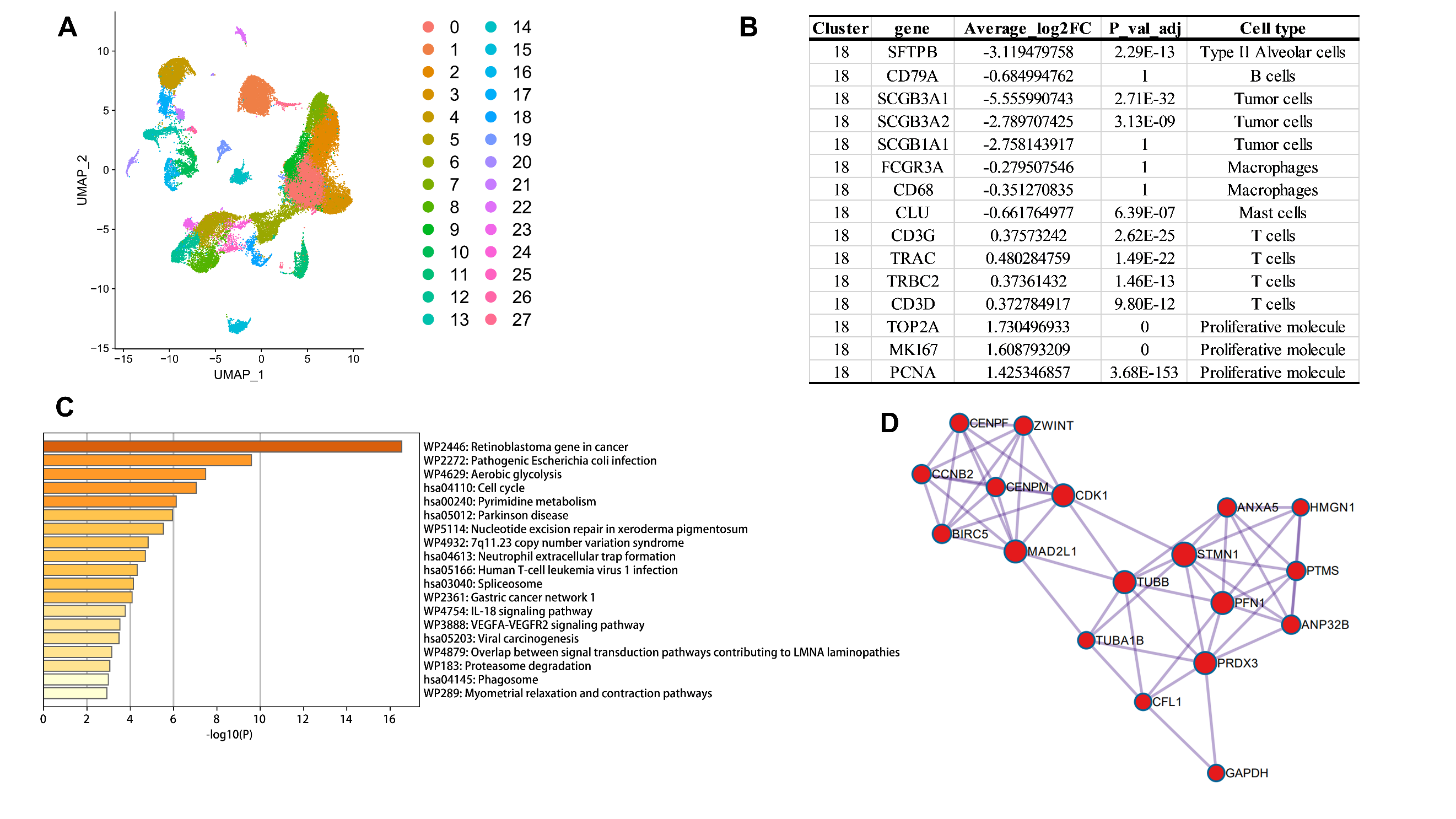


FIGURE S5. Pathway enrichment analysis of cell cluster expressing CCNB2 in LUAD.

(A) Uniform Manifold Approximation and Projection (UMAP) distribution of 28 cell clusters through unsupervised clustering.

(B) Parameters of cell clustering for cluster 18.

(C) Pathway enrichment analysis of upregulated genes on cluster 18.

(D) Mcode module of CCNB2 in upregulated genes on cluster 18.
